# Genetics of Waardenburg Syndrome in Africa: A Systematic Review

**DOI:** 10.3390/ijms27010127

**Published:** 2025-12-22

**Authors:** Elvis Twumasi Aboagye, Ramses Peigou Wonkam, Carmen de Kock, Collet Dandara, Ambroise Wonkam

**Affiliations:** 1 Department of Pathology, Division of Human Genetics, Faculty of Health Sciences, University of Cape Town, Cape Town 7925, South Africa or etaboagye@ug.edu.gh (E.T.A.); pgwram001@myuct.ac.za (R.P.W.); carmen.dekock@uct.ac.za (C.d.K.); collet.dandara@uct.ac.za (C.D.); 2 West African Centre for Cell Biology of Infectious Pathogens (WACCBIP), University of Ghana, Legon, Accra P.O. Box LG 54, Ghana; 3 Platform for Pharmacogenomics Research and Translation Unit (PREMED), South African Medical Research Council, Cape Town 7925, South Africa; 4 McKusick-Nathans Institute and Department of Genetic Medicine, Johns Hopkins University School of Medicine, Baltimore, MD 21205, USA

**Keywords:** Waardenburg syndrome, hearing loss, genetics, African populations

## Abstract

Waardenburg syndrome (WS) represents a group of genetic conditions characterized by auditory and pigmentation defects. Pathogenic variants in *PAX3*, *MITF*, *SOX10*, *EDN3*, *EDNRB*, *SNAI2*, and *KITLG* genes have been associated with WS across multiple populations; a comprehensive study of WS in Africa has not yet been reported. We conducted a systematic review of clinical expressions and genetics of WS across Africa. The Preferred Reporting Items for Systematic Reviews and Meta-Analysis (PRISMA) guidelines were followed, and the study protocol was registered on PROSPERO, the International Prospective Register of Systematic Reviews (2025 CRD420250655744). A literature search was performed on Google Scholar, PubMed, Scopus, Directory of Open Access Journals (DOAJ), Global Index Medicus, African-Wide Information, ScienceDirect, Connecting Repositories (CORE), and the Web of Science databases. We reviewed a total of 15 articles describing 84 WS cases, which showed no gender bias and a mean age at reporting of 17.5 years. Congenital, sensorineural, and profound hearing loss was described in most cases (66.7%; *n* = 56/84). WS type 2 (WS2), with characteristically no dystopia canthorum, is the predominant subtype (36.9%; *n* = 31/84). Pathogenic variants in four WS known genes, i.e., *PAX3* (13 families), *SOX10* (7 families), *EDNRB* (4 families), and *EDN3* (1 family), were reported in Morocco, Tunisia, and South Africa. One candidate gene (*PAX8*) was described in one family in Ghana. Two non-syndromic hearing loss (NSHL) genes (*BDP1* and *MYO6*) were reported in two separate families in South Africa, suggesting a possible phenotypic expansion. The highest number of WS cases was described in South Africa (38.1%; *n* = 32/84) and Tunisia (26.2%; *n* = 22/84). Gene variants were missense (27/43), deletion (7/43), splicing (5/43), nonsense (2/43), indel (1/43), and duplication (1/43), chiefly segregating in an autosomal dominant inheritance mode. There was no functional data to support the pathogenicity of putative causative variants. This review showed that WS2 is the most common in Africa. Variants in *PAX3* and *SOX10* were the predominant genetic causes. This study emphasizes the need to further investigate in-depth clinical characterization, molecular landscape, and the pathobiology of WS in Africa.

## 1. Introduction

Waardenburg syndrome (WS) comprises a group of heterogeneous genetic conditions, characterized by hearing loss (HL) and pigmentation abnormalities of the skin, hair, and eyes. WS is the most prevalent (2–5%) syndromic defect associated with congenital sensorineural HL [1,2,3]. Based on clinical features, WS can be classified into four types (WS1-4). Presence or absence of dystopia canthorum distinguishes WS1 (MIM# 193500) from WS2 (MIM# 193510; 600193; 606662; 608890; 611584). Limb anomalies differentiate WS3 (MIM# 148820) from WS1, while WS4 (MIM# 277580), also known as Shah–Waardenburg or Waardenburg–Hirschsprung syndrome, is defined by the presence of aganglionic megacolon. Despite this clinical heterogeneity, HL remains the most disabling feature affecting the well-being and quality of life of individuals with WS. 

Genetically, WS is equally heterogeneous. To date, pathogenic variants in six established genes (*PAX3*, *MITF*, *SOX10*, *EDN3*, *EDNRB*, and *SNAI2*) and two candidate genes (*KITLG* and *PAX8*) have been associated with the condition worldwide (https://hereditaryhearingloss.org/waardenburg, accessed on 30 June 2025). Over 400 WS-associated variants have been reported [4], and multiple genetic subtypes have been defined, particularly within WS2 (WS2A, B, C, D, and E) and WS4 (WS4A, B, and C). Nevertheless, the molecular underpinnings driving the variable clinical expressions and incomplete penetrance remain unclear. 

A prevailing biological explanation positions WS as a neurocristopathy arising from defects in the migration, proliferation, and differentiation of neural crest-derived cells [1,4,5]. The disruption of these developmental pathways affects melanocytes, craniofacial structures, limb musculature, and enteric ganglia, providing a unifying model for the multisystem phenotype. Alternatively, the characteristic craniofacial features have led authors to propose that WS may be a variation of branchial arch syndrome [6,7]. However, this hypothesis does not fully account for its broad range of clinical manifestations. 

While there is a fair amount of literature on WS from other continents, a comprehensive study of WS in Africa has not yet been reported. In this systematic review, we present the most comprehensive assessment to date of the phenotypic spectrum and genetic causes of WS in Africa.

## 2. Materials and Methods

We investigated all published articles reporting and describing WS phenotypes and associated genetic etiology from Africa, following the Preferred Reporting Items for Systematic Reviews and Meta-Analyses (PRISMA) guidelines [8]. We registered the study protocol and strategy for this review on PROSPERO, the International Prospective Register of Systematic Reviews, with the registration number under the following reference: 2025 CRD420250655744.

### 2.1. Literature Search Strategy

**Figure 1:**
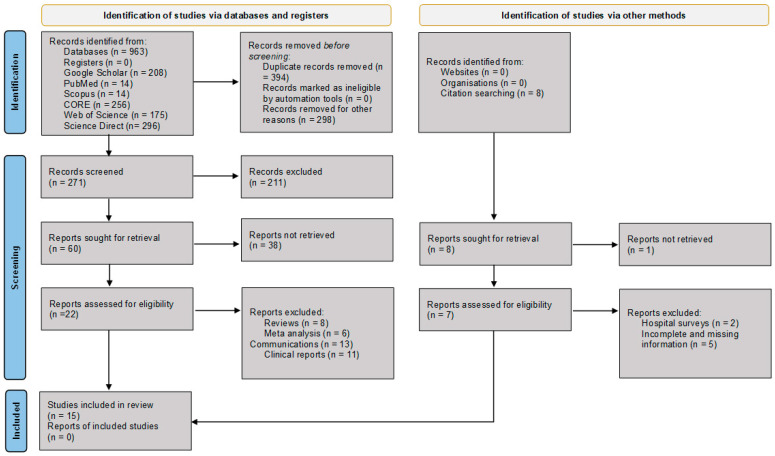
PRISMA flow diagram illustrating the identification, screening, eligibility assessment, and inclusion of studies for data extraction, synthesis, and analysis [8]. CORE = COnnecting REpositories.

We conducted an extensive electronic database search, including Google Scholar, PubMed, Scopus, Directory of Open Access Journals (DOAJ), Global Index Medicus, African-Wide Information, ScienceDirect, Connecting Repositories (CORE), and the Web of Science, for relevant articles. The search strictly used the following search string: Waardenburg Syndrome AND “Africa” AND “Genetics”, Waardenburg Syndrome AND “Africa” AND “Genetic Variants”, and Waardenburg Syndrome AND “Africa” AND “Genetic Variants” NOT “Review” in [Title AND/OR Abstract] separately. Zotero reference manager (https://www.zotero.org/) was used to build search results and generate a bibliography, and citations were retrieved from the search build folder. The PRISMA schematic flow chart guide was followed for the identification, screening, eligibility, and selection of included articles (Figure 1). The article search parameters covered WS disease-associated and/or causal variants reported in Africa. All cited references in the selected articles that met the outlined study inclusion criteria were checked, retrieved, and considered for synthesis until no further relevant studies were identified. 

### 2.2. Selection Criteria and Data Extraction

The literature search was conducted until 28 February 2025, with regular updates, considering only full-text articles in English for information retrieval. Study eligibility was assessed using the following inclusion criteria: HL case description with WS and/or WS-like clinical manifestations; and WS case with probable genetic etiology and/or associated with a likely pathogenic variant. In addition to removing duplicates, reviews and meta-analyses, policy documents, communications, articles reporting on non-African WS cases, and unrelated studies were systematically excluded, in line with the PRISMA guidelines, based on the title and abstract, as well as articles not published in English.

For selected articles, demographic variables (ethnic identity and origin, gender, and age at phenotype onset), clinical phenotypes (HL type, cause, severity, and laterality), and genetic characteristics (gene, variant, allele frequency, variant pathogenicity, variant classification tools used, genetic investigation strategy, variant analysis method, variant annotation, and filtration strategy) were captured. Reported disease-causing variants and WS-associated variants were manually curated using the American College of Medical Genomics (ACMG) [9] guidelines. Alternate allele frequency (AAF) of variants was queried on relevant population databases, including Bravo (https://bravo.sph.umich.edu/) and TOPMed (https://topmed.nhlbi.nih.gov/), accessed on 30 June 2025. 

Data extraction, quality assessment, and synthesis were conducted independently by the first and second authors of this manuscript (ETA and RPW). To avoid article selection bias, Sohani et al.’s quality of genetic studies (Q-Genie) [10], the Appraisal tool for Cross-Sectional Studies (AXIS tool) [11], Hoy et al.’s risk-of-bias assessment [12], and the PRISMA quality of meta-analysis tools were used (Tables S1–S4). Altogether, the relevance of each study’s outcome informed the final decision for selection. All discrepancies in data extraction, study selection, and synthesis were thoroughly discussed and resolved by all authors.

### 2.3. Data Analysis

Study-level characteristics (publication year, type of investigation, study design and method of examination, and first author), participant characteristics (number of families, number of individuals affected, age of WS onset, gender, population, and WS type; pigmentation disorders, dystopia canthorum, limbs defect, intestinal complications, and mobility disorder), HL features (type, severity, laterality, additional auditory report, and examination details), and genetic variant details (gene, transcript, variant (Human Genome Variation Society nomenclature), dbSNP reference SNP, protein change, pathobiology, penetrance, reported pathogenicity, inheritance mode, ACMG classification, in silico variant effect prediction tools scores, ClinVar, ClinGen, and Uniprot) were systematically extracted and analyzed. To examine molecular mechanisms underlying phenotypic variability and incomplete penetrance in WS, publicly available functional evidence for each implicated gene-disease pair was also reviewed.

For studies describing multiple individuals, only participants with sufficient phenotypic or genetic information were included in the analysis. All statistical calculations were performed using STATA 19 (StataCorp LLC, College Station, TX, USA).

## 3. Results

### 3.1. Article Search Results

Figure 1 illustrates the schematic flow chart of the article selection process. A total of 564 articles were initially identified from the title and abstract search. After removing duplicates and excluding non-English articles, non-African reports, and unrelated studies, 60 articles were considered for full-text review. Of these, 15 studies met the inclusion criteria and were selected for data extraction, synthesis, and analysis [13,14,15,16,17,18,19,20,21,22,23,24,25,26,27,28].

Of note, seven studies partially met the inclusion criteria but were excluded from analysis due to incomplete or missing clinical and genetic information. Most of these reports consisted of clinical surveys of deceased individuals presenting with WS-like features. One study described a probable founder variant in a three-generation family pedigree [29], while a related investigation reported 52 individuals with WS from 11 Southern African families but provided no individual-level clinical or molecular data [30]. Another survey of 3,006 deaf children identified 90 individuals presenting with WS features but lacked detailed phenotypic characterization [31]. A report describing a 9-year-old girl with WS and bilateral profound HL was excluded due to insufficient information [32]. Similarly, a prevalence study that described 13 WS cases with suspected autosomal dominant inheritance among black South Africans lacked adequate detail for inclusion [33]. A school-based survey of 240 deaf children identified 16 WS cases, but no phenotypic information was provided [34]. Another report describing 8 individuals with WS features among 366 deaf persons screened in Cape Town lacked essential clinical and genetic details and was therefore excluded [35]. Additional studies were excluded because full-text access was unavailable, although their titles suggested potential genetic contributions to WS in South Africa, Nigeria, and Kenya [36,37,38,39].

The 15 articles that met the study inclusion criteria described 84 WS cases from 57 families across ten (10) countries in Africa. Table 1 describes the WS participants reported in this article.

### 3.2. Biased and Quality Assessment

Risk-of-bias and quality assessments of the selected studies, evaluated using three independent tools, are presented in Tables S1–S3 [10,11,12]. Overall, the included studies demonstrated a low risk of bias.

### 3.3. Waardenburg Syndrome Participants’ Clinical Phenotypes

Across the 15 eligible studies, a total of 84 WS-affected participants were reported. Of these cases, 78.5% (*n* = 66/84) had gender information, with an equal distribution of males and females (50.0%; *n* = 33/66, Table 1). The mean age of participants was 17.5 years, with a standard deviation of ±12.9 (ages were available for 48/84 participants). 

**Figure 2:**
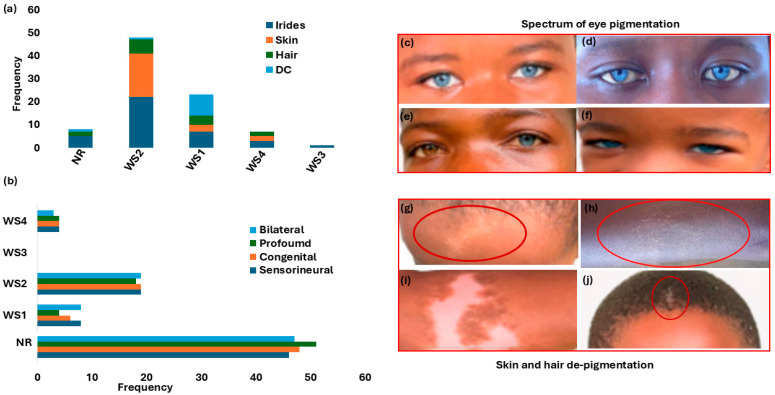
Waardenburg syndrome: characteristic features and spectrum of pigmentation presentations. Panel (**a**): Hearing loss phenotypes across WS types. Panel (**b**): Distribution of pigmentation abnormalities (iris, skin, and hair) and dystopia canthorum by WS type. (**c–f**) shows the array and shades of eyes, hair, and skin pigmentations. Panels (**c**,**d**,**f**) show bilateral blue eyes (hypochromic blue). Panel (**e**) shows unilateral complete heterochromia iridum (brown right eye and blue left eye). Panels (**g–i**) depict the depigmentation of skin WS feature presentations (forehead and chest, indicated with the red circles). Panel (**j**): Premature hair greying (indicated with the red circle). Figure 2c–j images were adapted from our open-access and previously published data (https://www.mdpi.com/2073-4425/16/3/257 and https://www.nature.com/articles/s42003-022-03326-8 accessed on 25 June 2025). WS = Waardenburg syndrome; NR = not reported; DC = dystopia canthorum.

Regarding auditory phenotype manifestations, sensorineural defects were the principal cause of HL (66.7%, *n* = 56/84). Two independent reports described conductive and mixed HL (1.2%; *n* = 1/84). The HL type was undetermined in a significant proportion of WS cases (30.9%; *n* = 26/84). Most reviewed cases (67.8%; *n* = 57/84) described congenital HL. One prelingual onset was reported, and 30.9% (*n* = 26/84) did not report any information on HL type (Figure 2a and Table 1). Bilateral HL was the most frequently observed laterality pattern (70.2%, *n* = 59/84), with unilateral HL reported in one case, while the remaining 28.5% (*n* = 24/84) had no laterality information. Although the severity of HL among cases was predominantly profound (60.7%; *n* = 51/84), a significant number of affected individuals’ degree of severity was undetermined (26.2%; *n* = 22/84, Table 1 and Figure 2b). None of the studies provided longitudinal follow-up, preventing the assessment of HL progression. 

### 3.4. Waardenburg Syndrome Subtype Classification

Figure 2a details the distribution of the different WS types, based on clinical presentations. WS2 (no dystopia canthorum) was the most reported (36.9%; *n* = 31/84). A significant proportion of participants had no data on WS classification type (44.0%; *n* = 37/84) (Figure 2a and Table 1).

The variable clinical expressions and incomplete penetrance, including degree of pigmentation defect (striking blue eyes, iris discoloration and heterochromia, premature greying, and skin depigmentation), are shown in Table 1 and Figure 2c–h. Iris discoloration was present in all WS types, characteristically described as sapphire-blue eyes in over 52% (44/84); 11.9% (10/84) as blue eyes; and the incidence of isolated bright blue eyes, segmented, sapphire, and complete blue eyes in separate reports (Table 1 and Figure 2c–h). Skin and hair pigmentation disorders were also reported in WS1, WS2, and WS4 (Table 1 and Figure 2g–j).

### 3.5. Waardenburg Syndrome Genetic Aetiology

**Figure 3:**
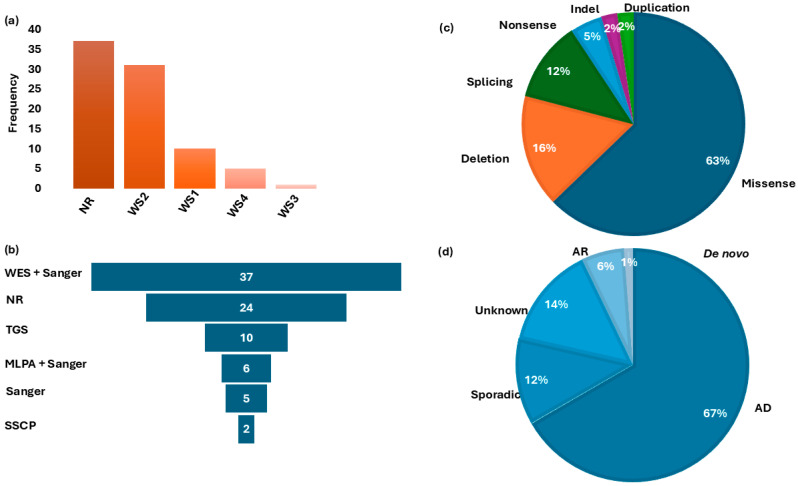
Waardenburg syndrome type distribution, Molecular investigation methods, gene variants classification, and mode of inheritance. Panel (**a**): Distribution of WS-type frequencies in studies reported in Africa. Panel (**b**) shows the frequency of different molecular methods used for genetic investigations. Panel (**c**): Distribution of associated gene variants. Panel (**d**): Classification of the pattern of inheritance in families reviewed. WS = Waardenburg syndrome; WES = whole-exome sequencing; NR = no report on molecular methods; TGS = targeted gene sequencing; MLPA = multiple ligation-dependent probe amplification; SSCP = single-strand conformation polymorphism; AD = autosomal dominant; AR = autosomal recessive; Indel = insertion/deletion.

Five distinct molecular methods were used across the included studies to investigate the genetic basis of WS (Figure 3a). Whole-exome sequencing (WES) followed by Sanger sequencing was the most commonly employed approach (44.0%; 37/84). Other molecular techniques (Figure 3b) included targeted gene sequencing (TGS) in 11.9% (*n* = 10/84), multiple ligation dependent probe amplification (MLPA) combined with Sanger sequencing (7.1%; *n* = 6/84), Sanger sequencing alone (5.9%; *n* = 5/84), and single-strand conformation polymorphism (SSCP) (2.4%; *n* = 2/84). WES investigations typically involved sequencing at least two affected individuals per family, together with first-degree relatives, followed by Sanger sequencing to confirm candidate variants and perform segregation analysis. TGS employed a 113-gene hearing loss panel sequenced on the Illumina HiSeq 2500 platform.

**Figure 4:**
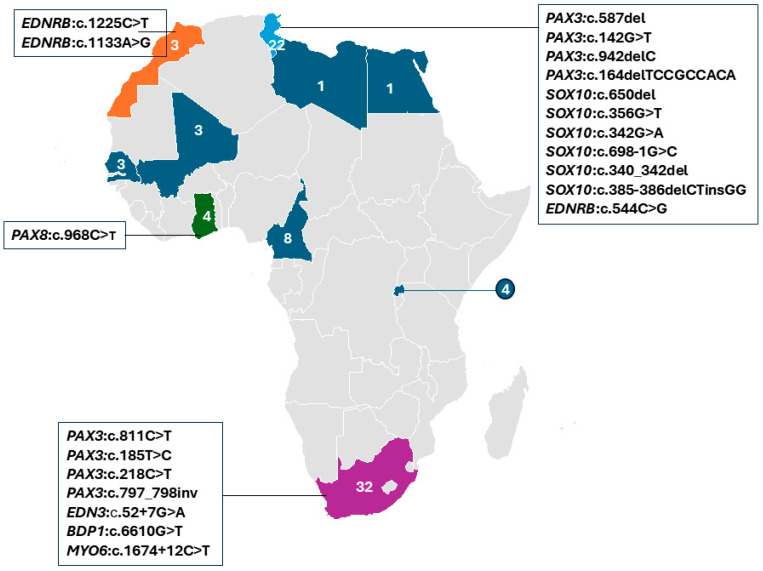
Distribution of reported WS studies by African country, with at least one report (colored). The specific genes and variants identified in affected individuals are indicated. The numbers show the summed reports of Waardenburg syndrome individuals in each country, while different colors are used to differentiate the countries.

South Africa accounted for the largest proportion of WS cases in the studies reviewed, with 38.1% (32/84), followed by Tunisia with 26.2% (22/84) of individuals (Figure 4). Putative genetic etiology was reported in 71.4% (60/84) of individual WS cases. Pathogenic variants were identified in four established WS genes, in a total of 26 families, i.e., *PAX3* (13 families), *SOX10* (7 families), *EDNRB* (4 families), and *EDN3* (1 family), which were reported in Morocco, Tunisia, and South Africa, respectively. Additionally, a candidate variant in *PAX8* was described in one family from Ghana (Figure 4; Table 2). Thus, *PAX3*-associated variants were frequently reported in 46.1% (12/26 families), followed by *SOX10* variants at 23.1% (6/26 families) and 15.3% (4/26) and 3.8% (1/26 families) in *EDNRB* and *EDN3,* respectively. In addition, variants in two non-syndromic hearing loss (NSHL) genes (*BDP1* and *MYO6*) were reported in two independent South African families with WS features, suggesting a possible phenotypic expansion. A total of 24 cases remained unresolved, including 13 simplex cases, 2 cases from one multiplex Ghanaian family, and 9 cases from five multiplex South African families, even after WES investigations.

Analysis of the reported genes against the WS types confirmed that WS1 and WS3 were primarily associated with *PAX3* variants, whereas WS2 was linked to *SOX10* variants, and WS4 cases were associated with both *EDNRB* and *SOX10* variants (Table 2). *PAX3* pathogenic variants were identified in both WS1 and WS3 cases. Pathogenic variants in *SOX10* were predominantly reported in WS2 and WS4 cases. *EDNRB* and *SOX10* variants were described exclusively in WS4 separately (Table 2). *EDNRB* variants were dominant in Morocco. 

Within these genes, likely causal variants were identified in 51.1% of the total WS cohort (43/84). Of these, 60.4% (26/43) were recurrent within countries and likely population-specific (Figure 4, Table 2). In addition, we found population-/country-specific variants, e.g., in PAX3 (Table 2), suggesting possible founder effects. As described in Figure 3c, the identified causal variants consisted of missense (27/43), deletion (7/43), splicing (5/43), nonsense (2/43), indel (1/43), and duplication (1/43) types. Observed segregation of phenotype with the identified genetic variants was mostly compatible with an AD pattern of inheritance (Figure 3d and Table 2). 

### 3.6. Manual Curation and Comparative Review of Targeted WS-Associated Variants

Manual curation of the reported variants revealed possible mutational hotspots at specific amino acid positions for some of the identified *PAX3* and *SOX10* variants in WS individuals. For example, at the *PAX3* (NM_181458.4):c.142G>T-p.(Gly48Cys) variant position, four previously reported pathogenic variants (p.Gly48fs, p.Gly48Ala, p.Gly48Arg, and p.Gly48Ser) that alter the same amino acid (a different nucleotide change in the codon) were identified (Table S5). In addition, *PAX3* (NM_181458.4):c.808G>C-p.(Arg270Gly), known as the WS1 pathogenic variant, affects a residue at which other pathogenic substitutions have been described in global cohorts, including p.(Arg270Cys), p.(Arg270His), p.(Arg270Pro), and p.(Arg270Ser). At amino acid position 271, c.811C>T-p.(Arg271Cys) identified in a WS1 case corresponds to the same position as other known *PAX3*-p.(Arg271Gly) and p.(Arg271His) pathogenic variants, which were previously described. Likewise, a variant that alters the amino acid codon in *SOX10*(NM_006941.4):c.385-386delCTinsGG-p.(Leu129Pro) was identified, and the previously reported *SOX10*-p.(Leu129Pro), a known WS pathogenic variant, occurred at the same position (Table S5). 

To further evaluate the biological significance of the reported disease-associated variants, we curated the pathogenicity of the reported gene variants using the ACMG guidelines and queried TopMED and Bravo databases (Table 1). The reported variant classification established 44.2% (19/43) as pathogenic, 30.2% (13/43) as likely pathogenic, 4.6% (2/43) as benign, and 20.9% (9/43) as a variant of uncertain significance (Table 2). Investigations to identify the likely WS causal variants were limited to in silico variant effect prediction tools and databases for putative variants prioritization and classification. Importantly, none of the studies conducted cell-based and/or in vivo functional experiments to support the pathogenicity of the identified variants.

Despite the limited knowledge on WS pathogenesis, the wide phenotypic spectrum has been primarily linked to the disruption of normal regulatory networks, which primarily compromise neural crest cell development and melanocyte differentiation [40]. WS-implicated genes likely share common interactive networks and pathways, which may support their biological significance in WS development. Collectively, WS genes are involved in molecular processes including melanocyte differentiation, neural crest development, transcription regulation, neural cell migration, and pigmentation [41]. A review of available functional and in silico data highlights *PAX3*-*SOX10*-*MITF* as central in the regulatory network under a hierarchical transcription control [42]. Early neural crest cell specifications have been demonstrated to be regulated tightly by *PAX3,* a key upstream transcription factor [43]. Whereas *SOX10* is involved in both neural crest delineation and melanocyte differentiation regulation, *MITF* modulates melanocyte development and pigmentation [44]. At the cellular level, *PAX3* and *SOX10* synergistic activation via physical interaction activates *MITF*. Coordinated by cooperative enhancement, *PAX3*-*SOX10* directly binds to a conserved regulatory element in the *MITF* promoter region, which has been demonstrated by co-immunoprecipitation [45].

On the other hand, the endothelial network interaction genes (*EDN3* and *EDNRB*) operate in parallel to this transcription regulatory network [46]. The successful binding of the ligand EDN3 to its receptor EDNRB affects the downstream cascade of signaling events critical for neural crest cells migration, which is essential in enteric nervous system development [46]. *Wnt*-signaling components are believed to be potential modulators of WS gene expression, culminating in phenotype variability (neural crest cells’ fate-dependent pathology) [47]. Post-transcriptional regulation mechanisms, including nonsense-mediated mRNA decay (NMD) [48], alternative splicing, and microRNA activity on WS genes’ expressivity and penetrance, likely lead to the inter-intra family phenotype heterogeneity observed. 

## 4. Discussion

This systematic review is the most comprehensive integrative clinical and genetic analysis of WS from data across Africa. Collectively, the reviewed studies highlight an urgent need for more extensive clinical assessment and detailed genetic characterization of individuals with HL and suspected WS features across the continent. Indeed, findings reveal substantial gaps in clinical documentation and subtype-specific diagnostic criteria, which are essential for accurate classification. Genetic characterizations of WS-associated variants are reported in just a few countries (10/54). There was, as expected, a high allelic and locus heterogeneity for established WS genes. Moreover, there were 24 unresolved cases, including some from six multiplex families following exome sequencing analysis, suggesting the high prospect for novel variants and candidate gene discovery in largely underexplored and highly genetically diverse African populations [15]. However, the high proportion of molecularly unsolved cases, particularly among individuals labelled as WS2—where the defining clinical features (prelingual sensorineural hearing loss and pigmentary anomalies) are inherently less specific than those of WS1, WS3, or WS4—likely reflects a combination of diagnostic imprecision, as attested by substantial gaps in clinical documentation; phenotypic overlap with other hearing loss entities; blended or dual genetic diagnoses (Mendelian or otherwise); and pathogenic variation in known WS genes that remains undetected by conventional first-tier testing strategies such as Sanger sequencing or capture/PCR-based short-read NGS.

Overall, the observations reported here are broadly consistent with global WS epidemiology [1,49]. Nevertheless, over 50% of WS individuals exhibited sensorineural, congenital, bilateral, and profound HL; however, comprehensive data on audiological assessment, including auditory brainstem response (ABR), radiological tests, and inner ear imaging recommended for thorough WS evaluation, were absent in the studies analyzed. The large fraction of participants lacking formal audiometry likely reflects systemic challenge, a severe shortage of audiologists, limited healthcare access, inadequate diagnostic equipment, and financial constraints across many African settings [50]. Sociocultural factors, including the stigma and neglect associated with HL and pigmentation anomalies characteristic of WS, may also deter affected individuals from seeking clinical evaluations [4,51]. Additionally, the complete absence of longitudinal audiological information on affected individuals further magnifies the reported challenge of one audiologist per million people in 78% of African countries, compared to 10 audiologists per million in 52% of countries across Europe [49]. 

WS2 is the most common subtype reported among participants reviewed, showing high inter- and intrafamilial phenotype variability and incomplete penetrance. This finding aligns with global patterns [1]. In contrast, WS3 remains rare, also consistent with previous reports [1,4]. Likewise, AD inheritance in 67% of cases analyzed conforms to the often-reported pathogenic variant segregation pattern in families with WS disorder globally [1]. However, variations in clinical expression and incomplete phenotype documentation undermine the reliability of subtype classification across African cases. These limitations highlight the potential utility of integrating genetic findings with clinical features in refining WS subtype diagnosis, particularly when clinical information is incomplete. Strengthening genetic medicine infrastructure, including clinical genetics services and molecular diagnostic capabilities, is therefore essential for accurate WS classification in Africa. 

Though the MLPA, TGS, and SSCP molecular investigation methods used are very specific and effective in identifying single-nucleotide polymorphisms (SNPs) and small indels (insertions or deletions), they lack the breadth of WES. Given the limitations of WES in detecting complex genomic rearrangements, future applications of whole-genome sequencing (WGS) may be critical for resolving these cases and uncovering novel disease mechanisms. Of the seven known WS genes (*PAX3*, *MITF*, *SNAI2, SOX10*, *EDNRB*, *EDN3*, and *KITLG* candidate genes), pathogenic variants in four WS genes (*PAX3*, *SOX10*, *EDNRB*, and *EDN3*) and one candidate WS gene (*PAX8*) were implicated in cases reviewed across 10 African countries. We also report possible mutation hotspots that could be important for molecular studies on WS globally. In addition, we found population-/country-specific variants, e.g., in *PAX3* (Table 2), suggesting possible founder effects that merit further investigations. If confirmed with larger sample sizes, such findings could enable the implementation of an affordable targeted variant diagnosis strategy in clinical practice. However, outside these localized contexts, high allelic heterogeneity remains the prevailing pattern in the other populations and settings reported in this study. WS cases associated with *PAX3* variants were categorized into WS1 mostly and, in some cases WS3, according to the clinical profile presented. WS3 is a more severe condition than WS1. Though moderately rare [1], a thorough clinical evaluation of the categorized WS1 and WS3 would have confirmed unclassified WS-type cases. For instance, evidence of musculoskeletal deformities of the upper limbs supports the WS3 diagnosis [4]. Likewise, *SOX10* pathogenic variants were identified in WS2 and WS4 cases. Interestingly, *MITF* variants, which were primarily associated with WS2 individuals in non-African populations [52,53], were not reported in cases analyzed. However, since WS2 shares other characteristic features with WS1, extensive genetic characterization in this group of individuals may have identified some cases associated with *MITF*. Moreover, in the WS4 cases associated with *EDNRB* and *EDN3* pathogenic variants, the characteristic presentation of Hirschsprung disease [54] caused by enteric nervous system disorder was inconclusive. 

WS etiology, described as a paradigmatic neural defect, culminates from disrupted hierarchies of transcription regulation and signaling pathways, supporting prior deleted promoter findings [55]. Disruption of transcription regulatory networks underpins neural crest cell development and melanocyte differentiation [54]. Neural crest disorders described in neurocristopathies have been linked to unusual proliferation, survival, migration, and/or differentiation of neural crest melanocytes, manifesting the spectrum of WS clinical presentations (HL and associated pigmentation anomalies) [56]. *SOX10* pathogenic variants are associated with more severe neural defects, such as olfactory bulb agenesis, frequent in Kallman and Waardenburg syndrome cases [47,57]. However, none of the neurological disorders common in patients with *SOX10* pathogenic variants were reported in individuals with WS4 that we reviewed. Components of the *Wnt*-signaling pathway also influence WS gene expression and contribute to phenotypic variability, particularly in *MITF*-associated WS2 manifestations [47]. The pathological molecular mechanisms implicating the reported dysregulation of neural crest cell fate specifications include NMD [47,58], alternative splicing [59], and microRNA regulation [60]. The splice-site mutations in *PAX3* and *SOX10* likely translate into isoforms via alternative splicing, affecting the paired domain and C-terminal end activation domain that impact regulatory function [59,61]. Exon skipping (synthesizing nonfunctional protein) and hidden splice site activation (exon truncation and intron addition) have also been described in an isolated WS case [62]. Future studies exploring patient-derived induced pluripotent stem cell (iPSC) models, epigenetic regulation profiling of crucial transcription networks, and transcription regulatory modifiers are imperative to generate empirical data on personalized networks and pathway interactions. 

## 5. Conclusions

This review showed that WS2 is common in Africa and reveals the lack of comprehensive clinical and in-depth molecular characterization in reported cases. We reported variants predominantly in *PAX3*, *SOX10*, *EDNRB*, and *EDN3,* mostly inherited in an autosomal dominant manner in the families investigated. Variants in *PAX3* and *SOX10* were the predominant genetic causes. The observed genetic and allelic heterogeneity, as well as intra-family wide variability in clinical expressions, is similar to what is reported in WS cases in most populations. However, in-depth molecular experimentation to support the biological significance of identified variants was absent. This study emphasizes the need to further investigate in-depth clinical characterization, molecular landscape, and the pathobiology of WS in Africa. These findings revealed the urgent need to harness opportunities for novel variants and candidate gene discoveries in largely unexplored and highly genetically diverse populations in Africa. 

## Figures and Tables

**Table 1:** Reviewed Waardenburg syndrome studies’ clinical parameter distribution in Africa.

Countries	Families		HL Phenotypes						WS Type	Pigmentation Disorders			DC	Reference
	References	Affected Individual	Gender	Age	HL Type	HL Onset	HL Severity	HL Laterality		Iris	Skin	Hair		
Egypt	fam1	1	m	20	sensorineural	congenital	profound	bilateral	WS2	sectoral	absent	absent	absent	[13]
Morocco	fam2	1	m	na	sensorineural	congenital	profound	bilateral	WS4	striking blue	yes	yes	absent	[14] *
	fam2	1	f	na	sensorineural	congenital	profound	bilateral	WS4	absent	absent	yes	absent	[14] *
Ghana	fam3	1	f	na	sensorineural	congenital	profound	bilateral	unclassified	striking blue	absent	yes	absent	[15] *
	fam4	1	f	na	sensorineural	congenital	profound	bilateral	unclassified	striking blue	absent	yes	absent	[15] *
	fam4	1	f	na	sensorineural	congenital	profound	bilateral	unclassified	absent	absent	absent	absent	[15] *
Senegal	fam5	1	m	na	sensorineural	congenital	profound	bilateral	WS2	sapphire-blue	yes	yes	absent	[16]
	fam6	1	m	na	sensorineural	congenital	profound	bilateral	WS2	sapphire-blue	yes	absent	absent	[16]
	fam6	1	m	na	sensorineural	congenital	profound	bilateral	WS2	absent	yes	yes	yes	[16]
	fam7	1	m	na	sensorineural	congenital	profound	bilateral	WS2	sapphire-blue	absent	absent	absent	[16]
	fam7	1	m	na	sensorineural	congenital	profound	bilateral	WS2	sapphire-blue	absent	yes	absent	[16]
	fam8	1	m	na	sensorineural	congenital	profound	bilateral	WS2	sapphire-blue	absent	absent	absent	[16]
Morocco	fam9	1	f	0.3	sensorineural	congenital	profound	na	WS4	hypoplastic	yes	yes	absent	[17] *
Cameroon	fam10	1	na	na	sensorineural	congenital	profound	bilateral	WS2	sapphire-blue	yes	yes	absent	[18]
	fam10	1	na	na	sensorineural	congenital	profound	bilateral	WS2	sapphire-blue	yes	yes	absent	[18]
	fam11	1	na	na	sensorineural	congenital	profound	bilateral	WS2	sapphire-blue	yes	yes	absent	[18]
	fam11	1	na	na	sensorineural	congenital	profound	bilateral	WS2	sapphire-blue	yes	yes	absent	[18]
Mali	fam12	1	m	13	sensorineural	congenital	profound	bilateral	WS1	bright blue	absent	absent	yes	[19]
	fam13	1	f	na	sensorineural	congenital	profound	bilateral	WS1	bright blue	absent	absent	yes	[20]
	fam13	1	m	na	sensorineural	congenital	profound	bilateral	WS2	bright blue	absent	absent	absent	[20]
Tunisia	fam14	1	na	3	sensorineural	congenital	severe	bilateral	WS2	bright blue	yes	absent	absent	[21] *
	fam15	1	na	9	sensorineural	congenital	na	na	WS1	absent	absent	absent	yes	[21] *
	fam16	1	na	11	undetermined	na	na	na	WS2	sapphire-blue	absent	yes	absent	[21] *
	fam17	1	na	11	undetermined	na	na	na	WS4	absent	absent	absent	absent	[21] *
	fam18	1	na	13	undetermined	na	na	na	WS3	sapphire-blue	absent	absent	absent	[21] *
	fam19	1	na	14	undetermined	na	na	na	WS2	sapphire-blue	absent	absent	absent	[21] *
	fam20	1	na	15	sensorineural	congenital	profound	bilateral	WS4	sapphire-blue	absent	absent	absent	[21] *
	fam21	1	na	18	undetermined	na	na	na	unclassified	sapphire-blue	absent	absent	absent	[21] *
	fam22	1	na	20	undetermined	na	na	na	unclassified	sapphire-blue	absent	absent	absent	[21] *
	fam23	1	na	22	sensorineural	congenital	severe	bilateral	WS2	sapphire-blue	yes	absent	absent	[21] *
	fam24	1	na	29	sensorineural	congenital	profound	bilateral	unclassified	absent	absent	absent	yes	[21] *
	fam25	1	na	53	sensorineural	congenital	severe	bilateral	WS2	sapphire-blue	yes	absent	absent	[21] *
	fam26	1	na	na	sensorineural	congenital	severe	bilateral	WS2	bright blue	yes	absent	absent	[21] *
	fam27	1	na	na	sensorineural	congenital	profound	bilateral	WS1	absent	absent	absent	yes	[21] *
Cameroon	fam28	1	m	14	sensorineural	congenital	profound	bilateral	WS2	sapphire-blue	yes	absent	absent	[22]
	fam29	1	m	7	sensorineural	congenital	profound	bilateral	WS2	complete blue	yes	absent	absent	[22]
	fam30	1	f	6	sensorineural	congenital	profound	bilateral	WS2	sapphire-blue	yes	absent	absent	[22]
	fam31	1	m	9	sensorineural	congenital	profound	bilateral	WS2	segment	yes	absent	absent	[22]
	fam32	1	f	25	sensorineural	congenital	severe	bilateral	WS2	sapphire-blue	yes	absent	absent	[22]
	fam33	1	f	12	sensorineural	congenital	profound	bilateral	WS2	sapphire-blue	yes	absent	absent	[22]
Libya	fam34	1	m	36	sensorineural	congenital	profound	bilateral	WS2	absent	yes	absent	absent	[23]
Tunisia	fam35	1	f	24	sensorineural	na	profound	bilateral	WS1	blue	yes	absent	yes	[24] *
	fam36	1	m	1	sensorineural	na	profound	bilateral	WS1	blue	yes	yes	yes	[24] *
	fam37	1	f	3	sensorineural	na	profound	bilateral	WS1	blue	absent	yes	yes	[24] *
	fam38	1	m	3	sensorineural	na	profound	bilateral	WS1	blue	yes	yes	yes	[24] *
	fam39	1	f	21	sensorineural	na	profound	bilateral	WS1	absent	absent	absent	yes	[24] *
	fam40	1	m	0.2	undetermined	na	na	bilateral	WS1	blue	absent	yes	absent	[24] *
Rwanda	fam41	1	m	na	sensorineural	congenital	profound	bilateral	WS2	blue	yes	absent	absent	[25]
	fam42	1	f	na	sensorineural	congenital	profound	bilateral	WS2	blue	yes	absent	absent	[25]
	fam42	1	f	na	sensorineural	congenital	profound	bilateral	WS2	blue	yes	absent	absent	[25]
South Africa	fam43	1	m	16	sensorineural	congenital	severe-profound	bilateral	unclassified	na	na	na	na	[27] *
	fam43	1	m	na	undetermined	na	mild-profound	na	unclassified	na	na	na	na	[27] *
	fam43	1	f	na	undetermined	na	na	na	unclassified	na	na	na	na	[27] *
	fam44	1	m	8	sensorineural	congenital	profound	bilateral	unclassified	na	na	na	na	[27] *
	fam44	1	f	11	sensorineural	congenital	profound	bilateral	unclassified	na	na	na	na	[27] *
	fam45	1	f	39	mixed	prelingual	profound	bilateral	unclassified	na	na	na	na	[27] *
	fam45	1	m	na	undetermined	na	na	na	unclassified	na	na	na	na	[27] *
	fam46	1	m	34	sensorineural	congenital	na	bilateral	unclassified	na	na	na	na	[27] *
	fam46	1	f	46	sensorineural	congenital	mild-moderate	bilateral	unclassified	na	na	na	na	[27] *
	fam46	1	f	24	undetermined	congenital	na	na	unclassified	na	na	na	na	[27] *
	fam47	1	m	4	sensorineural	congenital	profound	bilateral	unclassified	na	na	na	na	[27] *
	fam47	1	f	34	sensorineural	congenital	moderate- profound	bilateral	unclassified	na	na	na	na	[27] *
	fam48	1	m	9	sensorineural	congenital	profound	bilateral	unclassified	na	na	na	na	[27] *
	fam48	1	f	na	undetermined	na	na	na	unclassified	na	na	na	na	[27] *
	fam49	1	f	12	sensorineural	congenital	profound	unilateral	unclassified	na	na	na	na	[27] *
	fam49	1	f	na	undetermined	na	na	na	unclassified	na	na	na	na	[27] *
	fam49	1	f	na	undetermined	na	na	na	unclassified	na	na	na	na	[27] *
	fam50	1	f	6	undetermined	congenital	profound	bilateral	unclassified	na	na	na	na	[27] *
	fam50	1	m	4	undetermined	congenital	profound	bilateral	unclassified	na	na	na	na	[27] *
	fam51	1	m	10	conductive	congenital	moderate	bilateral	unclassified	na	na	na	na	[27] *
	fam51	1	m	na	undetermined	na	na	na	unclassified	na	na	na	na	[27] *
	fam52	1	m	10	sensorineural	congenital	profound	bilateral	unclassified	na	na	na	na	[27] *
	fam52	1	f	48	undetermined	congenital	na	na	unclassified	na	na	na	na	[27] *
	fam53	1	f	8	sensorineural	congenital	profound	bilateral	unclassified	na	na	na	na	[27] *
	fam53	1	f	na	undetermined	na	na	na	unclassified	na	na	na	na	[27] *
	fam53	1	m	na	undetermined	na	na	na	unclassified	na	na	na	na	[27] *
	fam54	1	m	14	sensorineural	congenital	profound	bilateral	unclassified	na	na	na	na	[27] *
	fam54	1	f	na	undetermined	na	na	na	unclassified	na	na	na	na	[27] *
	fam55	1	m	6	undetermined	congenital	profound	bilateral	unclassified	na	na	na	na	[27] *
	fam55	1	f	31	undetermined	na	na	na	unclassified	na	na	na	na	[27] *
	fam56	1	m	11	sensorineural	congenital	severe profound	bilateral	unclassified	na	na	na	na	[27] *
	fam56	1	f	36	undetermined	na	na	na	unclassified	na	na	na	na	[27] *
Tunisia	fam57	1	f	na	undetermined	na	profound	na	WS2	yes	absent	yes	absent	[26] *
	fam57	1	f	na	undetermined	na	profound	na	WS2	yes	yes	yes	absent	[26] *

m = Male; f = female; na = not available; * = genetic investigation; WS = Waardenburg syndrome; fam = family; DC = dystopia canthorum.

**Table 2:** Genetic characterization of reported variants among Waardenburg syndrome cases reported in Africa.

Population	Method	Gene	cDNA Change	Function	Protein Change	ACMG	Inheritance	WS Type	Reference
Egypt	na	na	na	na	na	na	de novo	WS2	[13]
Morocco	WES, Sanger	*EDNRB*	c.1225 C>T	missense	p.(Arg409Trp)	P	AD	WS4	[14] *
	WES, Sanger	*EDNRB*	c.1225 C>T	missense	p.(Arg409Trp)	P	AD	WS4	[14] *
Ghana	WES, Sanger	unsolved	na	na	na	na	AD	unclassified	[15] *
	WES, Sanger	*PAX8*	c.968C>G	missense	p.(Pro323Arg)	B	AD	unclassified	[15] *
	WES, Sanger	*PAX8*	c.968C>G	missense	p.(Pro323Arg)	B	AD	unclassified	[15] *
Senegal	na	na	na	na	na	na	sporadic	WS2	[16]
	na	na	na	na	na	na	AD	WS2	[16]
	na	na	na	na	na	na	AD	WS2	[16]
	na	na	na	na	na	na	AD	WS2	[16]
	na	na	na	na	na	na	AD	WS2	[16]
	na	na	na	na	na	na	AD	WS2	[16]
Morocco	Sanger	*EDNRB*	c.1133A>G	missense	p.(Asn378Ser)	P	AR	WS4	[17] *
Cameroon	na	na	na	na	na	na	unknown	WS2	[18]
	na	na	na	na	na	na	unknown	WS2	[18]
	na	na	na	na	na	na	unknown	WS2	[18]
	na	na	na	na	na	na	unknown	WS2	[18]
Mali	na	na	na	na	na	na	unknown	WS1	[19]
	na	na	na	na	na	na	AD	WS1	[20]
	na	na	na	na	na	na	AD	WS2	[20]
Tunisia	Sanger	*SOX10*	c.342G>A	nonsense	p.(Trp114Ter)	P	AD	WS2	[21] *
	Sanger	*PAX3*	c.142G>T	missense	p.(Gly48Cys)	LP	AD	WS1	[21] *
	TGS	*SOX10*	c.385-386delCTinsGG	indel	p.(Leu129Gly)	VUS	AD	WS2	[21] *
	TGS	*SOX10*	c.650del	deletion	p.(Pro217Glnfs69)	VUS	AD	WS4	[21] *
	TGS	*PAX3*	c.808C>G	missense	p.(Arg270Gly)	P	AD	WS3	[21] *
	TGS	*SOX10*	c.356G>T	missense	p.(Arg119Leu)	P	AD	WS2	[21] *
	TGS	*SOX10*	c.698-1G>C	splice acceptor	na	P	AD	WS4	[21] *
	TGS	unsolved	na	na	na	na	unknown	unclassified	[21] *
	TGS	unsolved	na	na	na	na	unknown	unclassified	[21] *
	TGS	*SOX10*	c.340_342del	deletion	p.(Trp114del)	LP	AD	WS2	[21] *
	TGS	unsolved	na	na	na	na	unknown	unclassified	[21] *
	TGS	*SOX10*	c.340_342del	deletion	p.(Trp114del)	LP	AD	WS2	[21] *
	Sanger	*SOX10*	c.342G>A	nonsense	p.(Trp114Ter)	P	AD	WS2	[21] *
	Sanger	*PAX3*	c.142G>T	missense	p.(Gly48Cys)	P	AD	WS1	[21] *
Cameroon	na	na	na	na	na	na	AD	WS2	[22]
	na	na	na	na	na	na	sporadic	WS2	[22]
	na	na	na	na	na	na	AD	WS2	[22]
	na	na	na	na	na	na	sporadic	WS2	[22]
	na	na	na	na	na	na	sporadic	WS2	[22]
	na	na	na	na	na	na	sporadic	WS2	[22]
Libya	na	na	na	na	na	na	unknown	WS2	[23]
Tunisia	Sanger, MLPA	*PAX3*	c.942delC	deletion	p.(Pro314ProfsX6)	LP	AD	WS1	[24] *
	Sanger, MLPA	*PAX3*	c.942delC	deletion	p.(Pro314ProfsX6)	LP	sporadic	WS1	[24] *
	Sanger, MLPA	*PAX3*	c.587del	deletion	?	VUS	sporadic	WS1	[24] *
	Sanger, MLPA	*PAX3*	c.933_936dupTTAC	duplication	p.(Gln313LeufsX98)	LP	sporadic	WS1	[24] *
	Sanger, MLPA	*PAX3*	na	na	na	na	sporadic	WS1	[24] *
	Sanger, MLPA	*PAX3*	c.164delTCCGCCACA	deletion	p.(Ile55_His57del)	LP	sporadic	WS1	[24] *
Rwanda	na	na	na	na	na	na	unknown	WS2	[25]
	na	na	na	na	na	na	unknown	WS2	[25]
	na	na	na	na	na	na	unknown	WS2	[25]
South Africa	WES, Sanger	unsolved	*na*	na	na	na	AD	unclassified	[27] *
	WES, Sanger	unsolved	*na*	na	na	na	AD	unclassified	[27] *
	WES, Sanger	unsolved	*na*	na	na	na	AD	unclassified	[27] *
	WES, Sanger	*EDNRB*	c.764G>A	missense	p.(Cys255Tyr)	P	AD	unclassified	[27] *
	WES, Sanger	*EDNRB*	c.764G>A	missense	p.(Cys255Tyr)	P	AD	unclassified	[27] *
	WES, Sanger	*PAX3*	c.811C>T	missense	p.(Arg271Cys)	P	AD	unclassified	[27] *
	WES, Sanger	*PAX3*	c.811C>T	missense	p.(Arg271Cys)	P	AD	unclassified	[27] *
	WES, Sanger	*PAX3*	c.811C>T	missense	p.(Arg271Cys)	P	AD	unclassified	[27] *
	WES, Sanger	*PAX3*	c.811C>T	missense	p.(Arg271Cys)	P	AD	unclassified	[27] *
	WES, Sanger	*PAX3*	c.811C>T	missense	p.(Arg271Cys)	P	AD	unclassified	[27] *
	WES, Sanger	unsolved	na	na	na	na	AD	unclassified	[27] *
	WES, Sanger	unsolved	na	na	na	na	AD	unclassified	[27] *
	WES, Sanger	*EDN3*	c.52+7G>A	splicing	p.?	VUS	AD	unclassified	[27] *
	WES, Sanger	*EDN3*	c.52+7G>A	splicing	p.?	VUS	AD	unclassified	[27] *
	WES, Sanger	unsolved	na	na	na	na	AD	unclassified	[27] *
	WES, Sanger	unsolved	na	na	na	na	AD	unclassified	[27] *
	WES, Sanger	unsolved	na	na	na	na	AD	unclassified	[27] *
	WES, Sanger	*BDP1*	c.6610G>T	missense	p.(Val2204Phe)	VUS	AR	unclassified	[27] *
	WES, Sanger	*BDP1*	c.6610G>T	missense	p.(Val2204Phe)	VUS	AR	unclassified	[27] *
	WES, Sanger	*MYO6*	c.1674+12C>T	splicing	p.(=)	VUS	AD	unclassified	[27] *
	WES, Sanger	*MYO6*	c.1674+12C>T	splicing	p.(=)	VUS	AD	unclassified	[27] *
	WES, Sanger	*PAX3*	c.185T>C	missense	p.(Met62Thr)	LP	AD	unclassified	[27] *
	WES, Sanger	*PAX3*	c.185T>C	missense	p.(Met62Thr)	LP	AD	unclassified	[27] *
	WES, Sanger	*PAX3*	c.218C>T	missense	p.(Ser73Leu)	P	AD	unclassified	[27] *
	WES, Sanger	*PAX3*	c.218C>T	missense	p.(Ser73Leu)	P	AD	unclassified	[27] *
	WES, Sanger	*PAX3*	c.218C>T	missense	p.(Ser73Leu)	P	AD	unclassified	[27] *
	WES, Sanger	*PAX3*	c.797_798inv	missense	p.(Trp266Ser)	LP	AD	unclassified	[27] *
	WES, Sanger	*PAX3*	c.797_798inv	missense	p.(Trp266Ser)	LP	AD	unclassified	[27] *
	WES, Sanger	unsolved	na	na	na	na	AD	unclassified	[27] *
	WES, Sanger	unsolved	na	na	na	na	AD	unclassified	[27] *
	WES, Sanger	unsolved	na	na	na	na	AD	unclassified	[27] *
	WES, Sanger	unsolved	na	na	na	na	AD	unclassified	[27] *
Tunisia	SSCP	*EDNRB*	c.544C>G	missense	p.(Ala183Gly)	LP	AR	WS2	[26] *
	SSCP	*EDNRB*	c.544C>G	missense	p.(Ala183Gly)	LP	AR	WS2	[26] *

WES = Whole-exome sequencing; na = not available; P = pathogenic; LP = likely pathogenic; VUS = variant of uncertain significance; B = benign; AR = autosomal recessive; AD = autosomal dominant; ACMG = American College of Medical Genetics and Genomics; WS = Waardenburg syndrome; TGS = targeted gene sequencing (113 hearing loss-related loci); * = genetic investigation.

## Data Availability

No new data were created or analyzed in this study. Data sharing is not applicable.

## Supplementary Materials

The following supporting information can be downloaded at https://www.mdpi.com/article/10.3390/ijms27010127/s1.

## Abbreviations

The following abbreviations are used in this manuscript:

AAF	Alternate allele frequency;
ABR	Auditory brainstem response;
ACMG	American College of Medicine Genetics and Genomics;
AD	Autosomal dominant;
*BDPI*	B double prime 1;
CORE	Connecting repositories;
DC	Dystopia canthorum;
DOAJ	Directory of open access journals;
*EDN3*	Endothelin 3;
*EDNRB*	Endothelin receptor type B;
HL	Hearing loss;
*KITLG*	Kit ligand;
*MITF*	Melanocyte-inducing transcription factor;
MLDPA	Multiplex ligation-dependent amplification;
*MYO6*	Myosin VI;
*PAX3*	Paired box 3;
*PAX8*	Paired box 8;
PRISMA	Preferred Reporting Items for Systematic reviews and Meta-Analyses;
RNA	Ribonucleic acid;
*SNAI2*	Snail family transcription repressor 2;
SNPs	Single nucleotide polymorphisms;
*SOX10*	SRY-box transcription factor 10;
SSCP	Single-strand conformation polymorphism polymerase chain reaction;
TGS	Targeted gene sequencing;
WES	Whole-exome sequencing;
WS	Waardenburg syndrome.
